# The Feasibility of AgileNudge+ Software to Facilitate Positive Behavioral Change: Mixed Methods Design

**DOI:** 10.2196/57390

**Published:** 2024-11-13

**Authors:** Fereshtehossadat Shojaei, Fatemehalsadat Shojaei, Archita P Desai, Emily Long, Jade Mehta, Nicole R Fowler, Richard J Holden, Eric S Orman, Malaz Boustani

**Affiliations:** 1 Luddy School of Informatics, Computing, and Engineering Indiana University Bloomington Bloomington, IN United States; 2 School of Computer Science State University of New York Oswego, NY United States; 3 Center for Health Innovation and Implementation Science School of Medicine Indiana University Indianapolis, IN United States; 4 Division of Gastroenterology and Hepatology Department of Medicine Indiana University School of Medicine Indianapolis, IN United States; 5 Department of Medicine School of Medicine Indiana University Indianapolis, IN United States; 6 Center for Aging Research Regenstrief Institute, Inc Indianapolis, IN United States; 7 School of Public Health Indiana University Bloomington Bloomington, IN United States; 8 Sandra Eskenazi Center for Brain Care Innovation Eskenazi Health Indianapolis, IN United States

**Keywords:** AgileNudge+, agile, nudge strategy, nudging interventions, agile implementation, human behavior, software design, human-computer interaction, user experience design, usability testing

## Abstract

**Background:**

In today’s digital age, web-based apps have become integral to daily life, driving transformative shifts in human behavior. “AgileNudge+” (Indiana University Center for Health Innovation and Implementation Science) is a web-based solution to simplify the process of positive behavior change using nudging as an intervention. By integrating knowledge from behavioral economics with technology, AgileNudge+ organizes multiple steps, simplifies complex tasks, minimizes errors by enhancing user engagement, and provides resources for creating and testing nudge interventions.

**Objective:**

This paper aimed to outline the design process, methodologies, and usefulness of “AgileNudge+” for the development of evidence-based nudges. It used a mixed methods approach to evaluate the software’s interface usability and usefulness for creating and testing nudge interventions.

**Methods:**

AgileNudge+ was developed through iterative processes integrating principles from behavioral economics and user-centered design. The content of AgileNudge+ operationalizes an Agile science–based process to efficiently design, embed, and disseminate evidence-based nudges that encourage positive behavior change without limiting choice. Using a mixed methods approach, we tested AgileNudge+ software’s ability to organize and simplify the nudge intervention process, allowing a diverse range of scholars with limited knowledge of Agile science to use nudges. Usability testing assessed the tool’s usefulness and interface with a sample of 18 health care professionals, each asked to interact with the software and create a nudge intervention to solve a problem within their professional project’s sphere.

**Results:**

The study was funded in August 2022, with data collection occurring from June 2023 to July 2024. As of July 2024, we have enrolled 18 participants. Quantitative results found a mean usefulness rating of AgileNudge+ of 3.83 (95% CI 3.00-4.66). Qualitative results highlighted ways to modify the language used in AgileNudge+ to be more comprehensible to a diverse user base and promoted modifications to the software that facilitate real-time assistance and prioritize time efficiency in user interactions. Feedback further supported the positive impact of gamification on participant motivation when using the software.

**Conclusions:**

AgileNudge+ is an effective assistive tool for simplifying the positive behavior change process using nudge interventions, with tailored content and interactions to meet users’ needs and demands. Building onto the current design, future iterations of AgileNudge+ will use artificial intelligence to process large volumes of data while reducing the time and mental energy required to scan for existing cognitive biases and nudge prototypes. The software is also being upgraded to build on current gamification efforts, encouraging more sustained motivation by increasing the temporal resolution of the digital interface. These modifications stay true to the agility and user-centered aspects of AgileNudge+, emphasizing the novelty of the constantly evolving software design process.

## Introduction

Successful implementation of evidence-based health care solutions is critical for improving the safety, quality, and cost of health care services leading to better health outcomes [1,2]. However, successful implementation requires understanding, predicting, and changing the behaviors of individual human members and the overall complex adaptive health care delivery network. This desired behavior change can be spurred using Agile science [3-5]. Agile science is a process for knowledge discovery and acquisition that uses insights from behavioral economics, complexity science, and network science to develop context-sensitive and scalable tools, processes, and strategies to discover, implement, and diffuse evidence-based health care solutions within complex adaptive human networks. Because Agile science integrates multiple disciplines that seek to understand the decision-making within a larger system, operationalization of this knowledge can change the behavior of health care individuals or organizations to maximize the adoption of evidence-based solutions.

Agile science focuses on the psychological, cognitive, emotional, social, and cultural factors that influence the decisions of individuals living in dynamic social organizations. Individuals in these ever-changing social structures face high levels of uncertainty and require rapid decision-making [6]. Within Agile science, behavior change interventions are informed by dual process theory’s system-1 and system-2 framework [7]. In this framework, system 2 is a slow and deliberate information processing system that consumes a high level of conscious attention and effort [7]. In contrast, system 1 is an intuitive, fast, and automatic information process that relies on heuristic shortcuts [7]. By leveraging cognitive heuristics, such as anchoring, framing, or social proof, environmental adjustments can encourage specific choices [8]. Choice architecture refers to the modification of a physical, digital, or social environment where interpersonal human interactions or interactions with the surrounding environment take place. Environmental modifications can be specifically designed to overlap with the heuristic pathways used by system 1 to facilitate changes in human behavior, a process referred to as nudging [9].

Nudges are low-cost and scalable choice architectures that leverage system-1 processing to deliberately modify the social, physical, or digital environment, promoting behavioral change without forbidding choice [8,10]. Due to their affordable and scalable nature, designing, implementing, or diffusing evidence-based nudges are cost-effective processes for behavioral change [8,10]. Systematic reviews of nudges in health care settings found that 79% (19/24) of nudges lead to improvements in the quality of care without any unintended consequences [11]. Nudge benefits have been found to range from optimized medication prescription practices in line with evidence-based guidelines to increased hand hygiene compliance among clinicians, reducing the number of preventable infections [12,13]. Nudging effectively maximizes techniques to address behavioral determinants [12,13]. Examples of nudges include changing the default settings of physician ordering templates or electronic health records, introducing posters as tangible point-of-decision reminders, and modifying chair placement to subliminally encourage physicians to sit by the bedside and practice etiquette-based care [14-16]. Nudges can involve psychological rewards, provide economic-driven benefits, or play into humanistic tendencies to do good, which can increase willingness to bring medications to primary care visits, increase appointment scheduling in patient portals, or increase participation in research studies that longitudinally track health data [17-19]. Crafting an effective nudge requires mapping the digital, physical, and social environments; denudging any problem behavior; targeting specific cognitive heuristics; identifying any existing misaligned nudges; developing clear measurable goals with termination plans for failure; and performing rapid sprints to test the minimally viable nudge (Multimedia Appendix 1). The agility portion of nudge design, implementation, and diffusion comes from running sprints or iterative cycles of testing and modification. Agile processes allocate 90% of the time, social, financial, and emotional capital resources toward doing and testing, while only 10% of the time is allocated for planning, reflecting, and adjusting (Multimedia Appendix 1). These techniques emphasize context-specific implementation, increasing efficiency by not striving for perfection during the context-independent planning phases but instead monitoring and adapting the nudge to the environment it is embedded within. Ideal nudges are easy, attractive, social, and timely, and they are intentional about the messenger, incentives, norms, defaults, saliency, priming, affect, commitments, and ego cognitive heuristics (Multimedia Appendix 2).

Previous initiatives have focused on training dedicated professionals to become capable of designing, implementing, and diffusing evidence-based nudges [20]. For example, Indiana University School of Medicine’s Center for Health Innovation and Implementation Science (CHIIS) has provided a range of educational programs since its inception in 2013, including a year-long graduate certificate program. More recently, CHIIS partnered with the Indiana University School of Public Health-Bloomington to launch the Agile Nudge University to train individuals on applying Agile science to create, implement, and test nudges [20,21]. The US General Services Administration has also trained personnel that apply behavioral science concepts, such as nudges, to evaluate current program interventions and policy decisions [22]. Unfortunately, mastery in Agile nudge design, implementation, and diffusion takes time and financial resources, limiting the reach of educational programs.

To make these processes more accessible, we designed AgileNudge+, a web-based software app tailored to support health care system professionals who want to design, implement, and diffuse evidence-based nudges to facilitate behavioral change. AgileNudge+ would allow a diverse range of health care professionals to improve the performance of their complex adaptive health care delivery organization without being constrained by geographical limitations or requiring years of training to gain expertise before being able to implement a successful nudge. Agile science was used to develop the software and is a component of the nudge design process in the software, ensuring the software is appropriately tailored to health care professionals and to maximize the efficiency and scalability of the behavioral nudges that are designed. This study aims to examine how AgileNudge+ fosters behavioral change among health care professionals or researchers by using customized nudges and sprint cycles to enhance the performance of complex health care delivery systems.

## Methods

### Overview

The design of AgileNudge+ included 2 phases, that is, content development and the creation of steps within the software. The content is based off the Agile innovation, implementation, and diffusion processes developed by scientists at CHIIS [10] (Multimedia Appendix 1). The design of the software’s steps was informed by a co-design and design thinking process, proven effective in previous research [23]. This phase used week-long sprints to develop minimally viable prototypes, which were then presented in co-design sessions with stakeholders and evaluated for further refinement in the subsequent week. The co-design process, spanning 12 weeks, resulted in the creation of user personas, mapping user journeys, generating design concepts, and developing high-fidelity minimally viable prototypes.

### User Persona

**Figure 1:**
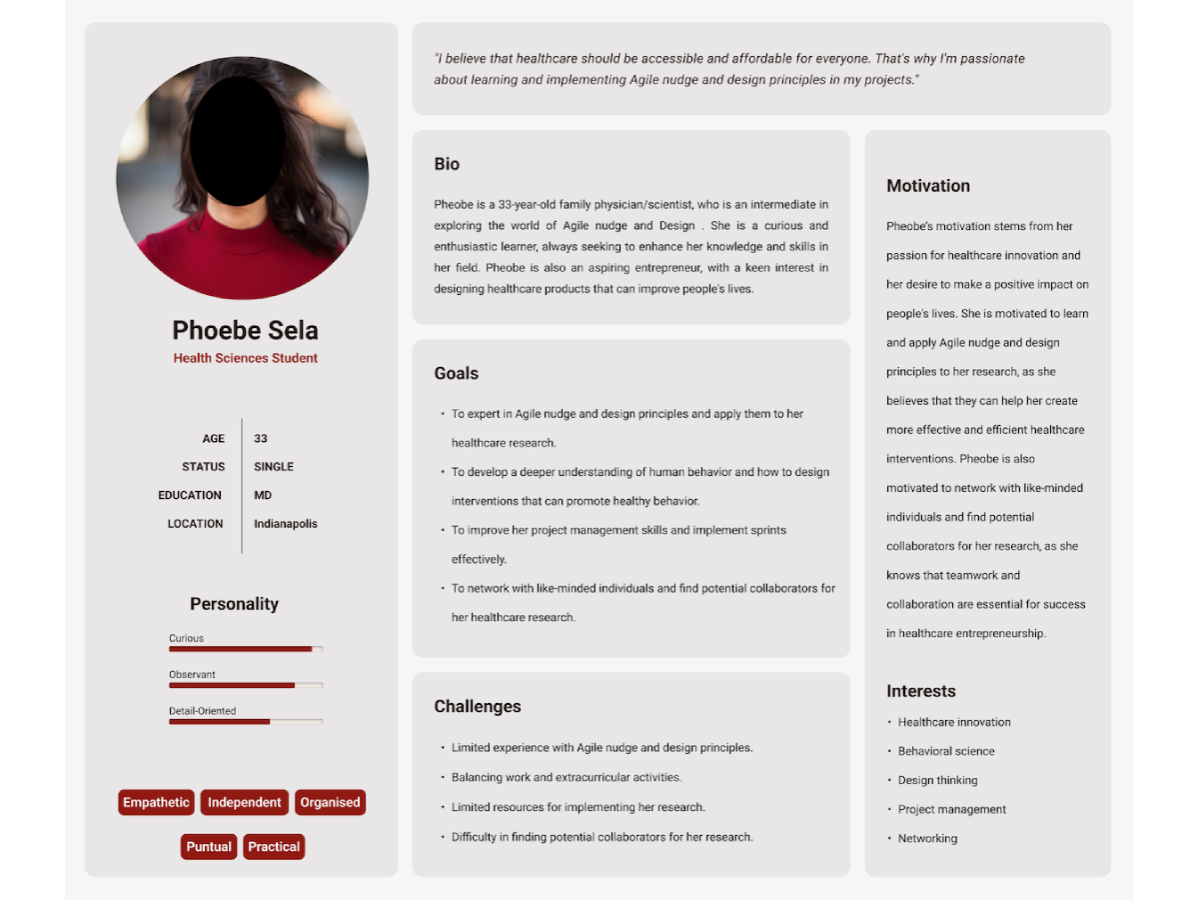
Phoebe Sela’s persona: this image illustrates the journey map of the persona used in the development of AgileNudge+, to ensure a user-friendly design that aligns with the expectations of individual users.

Personas are valuable tools for designers, creating a consistent understanding of users by representing their common behavioral traits and serving as a theoretically minimal viable prototype. By focusing on personas, designers can prioritize essential features and address specific needs, ensuring that the product aligns with user requirements [24-26]. In our project, we developed a persona named Phoebe, a 33-year-old family physician and scientist who aims to integrate Agile science into her profession to drive behavioral changes (Figure 1). Phoebe was crafted based on real-world insights, capturing her goals and challenges related to applying Agile science. Using Phoebe as our persona ensured that our design decisions were aligned with the needs and expectations of users like her, ultimately leading to a more user-centered and effective solution.

### User Journey Map

**Figure 2:**
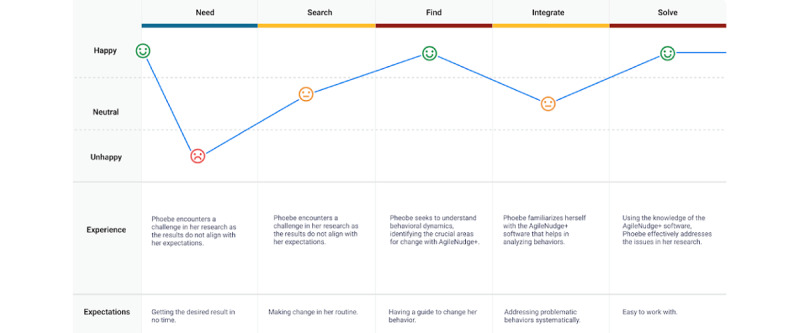
Phoebe Sela’s journey map: this journey map illustrates Phoebe’s interactions with the software, highlighting the challenges she may encounter, and her expectations for real-time behavioral change using AgileNudge+.

Following the creation of the persona, a user journey map was developed for Phoebe (Figure 2). This map illustrates the user’s interaction with the product across multiple steps, identifying areas where further user research is needed to enhance the user experience [27-29]. Creating Phoebe’s user journey map began with holding meetings with stakeholders to outline user’s goals and pain points with existing Agile innovation, implementation, and diffusion processes developed by scientists at CHIIS (Multimedia Appendix 1). Phoebe’s journey further illustrates her initial aspirations in health care research, navigating obstacles, exploring Agile science principles, integrating software, ultimately leading to transformative success.

### Must Have, Should Have, Could Have, and Won’t Have This Time Method

**Figure 3:**
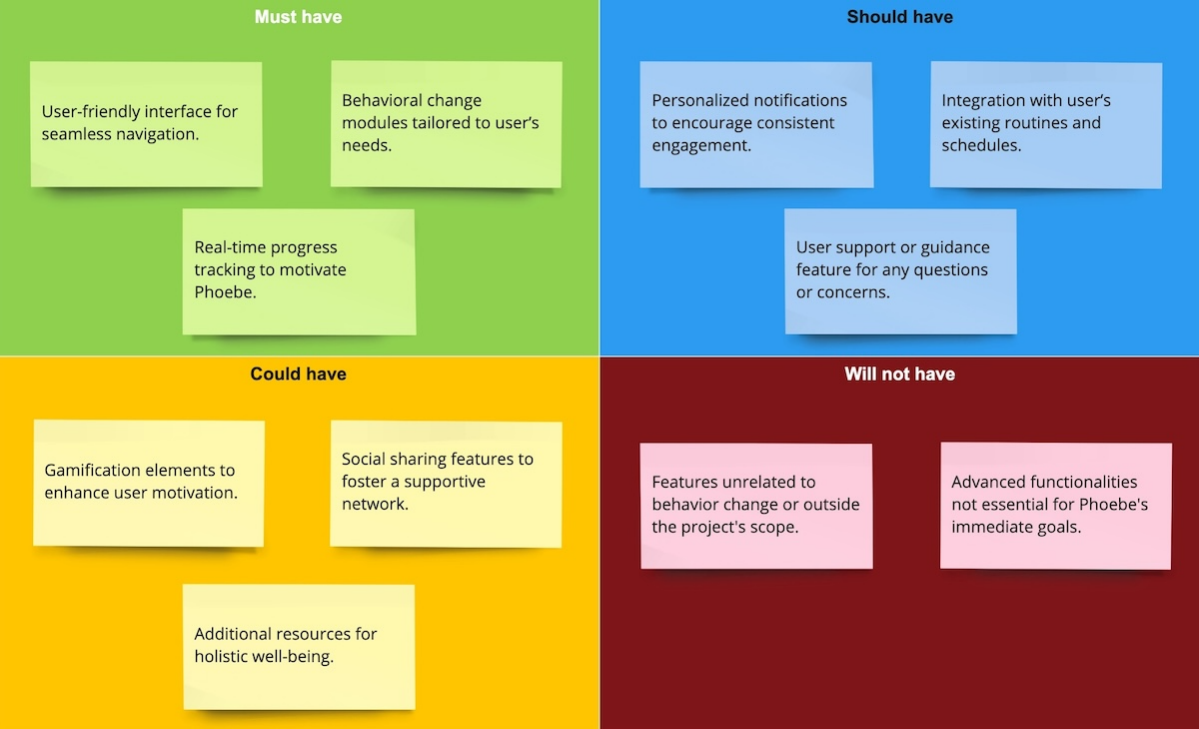
MoSCoW prioritization chart: this chart illustrates the user-centered design methodology that was implemented for the persona. MoSCoW: Must Have, Should Have, Could Have, and Won’t Have This Time.

The subsequent stage of the user-centered design methodology was to prioritize the needs, objectives, and satisfaction of end users while considering their ease of understanding and task completion [30]. In alignment with this methodology, we implemented the MoSCoW (Must Have, Should Have, Could Have, and Won’t Have This Time) method for the persona (Figure 3). The MoSCoW method categorizes requirements into “Must have,” “Should have,” “Could have,” and “Won’t have.” “Must have” requirements are indispensable for meeting business needs and are crucial for project success. “Should have” requirements are desirable but not critical to project success, while “Could have” requirements are considered nice-to-haves, and “Won’t have” requirements have varying degrees of priority that may be addressed in future developmental iterations or features that the business should avoid adding to the project [31].

### Minimally Viable Prototyping

**Figure 4:**
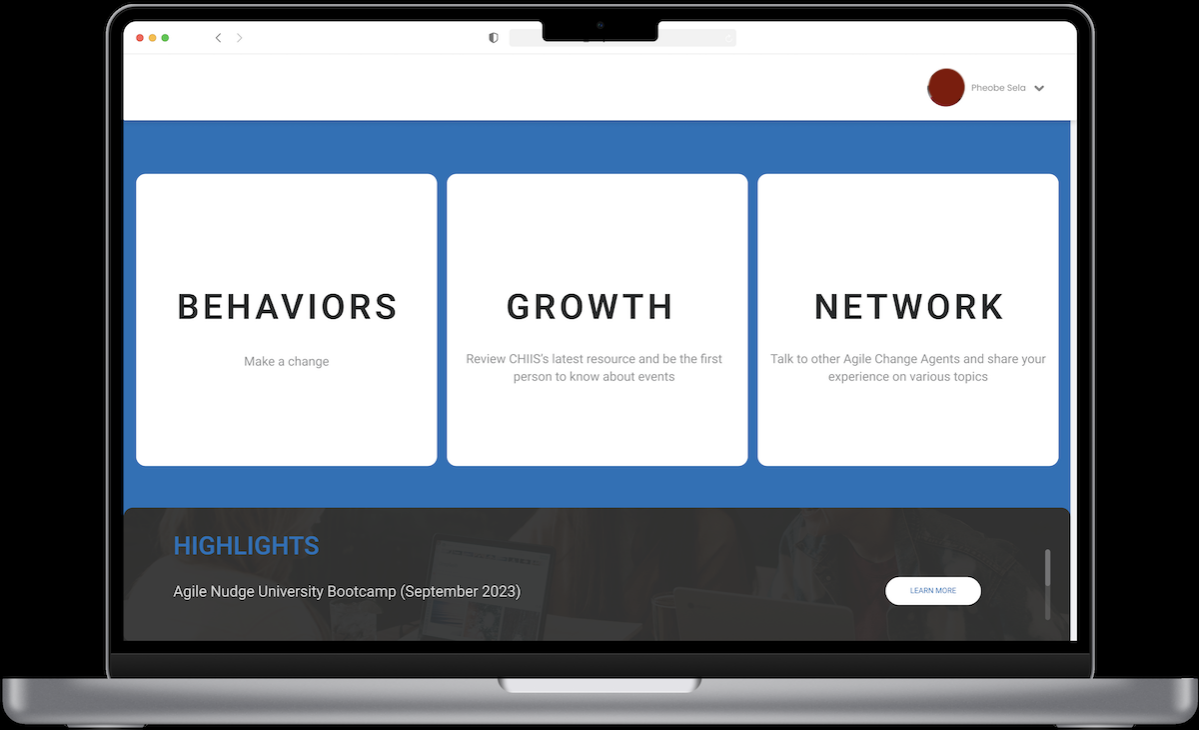
The AgileNudge+ homepage: this screen appears when users enter the URL for AgileNudge+. It allows users to access the latest resources, connect with other Agile Change Agents, and initiate a new behavioral change. CHIIS: Center for Health Innovation and Implementation Science.

**Figure 5:**
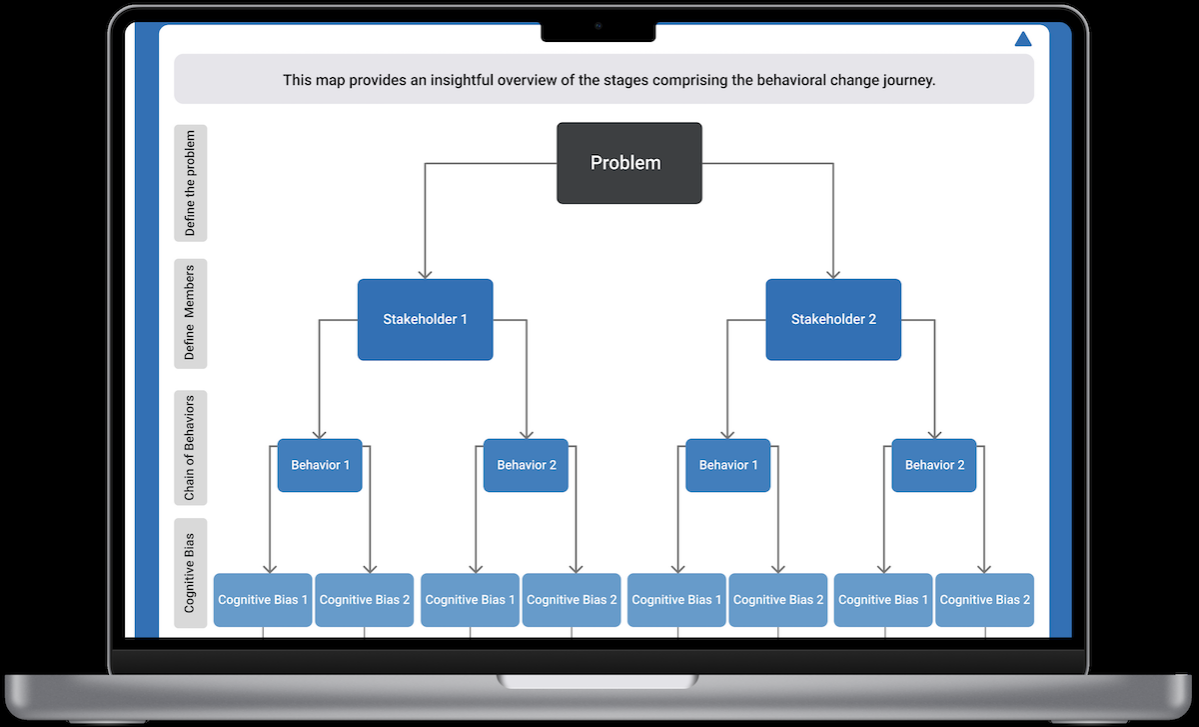
The AgileNudge+ behavioral change journey’s generated overview map: this screen is what users see once they input their problem, stakeholders, cognitive biases, and associated behavioral changes.

**Figure 6:**
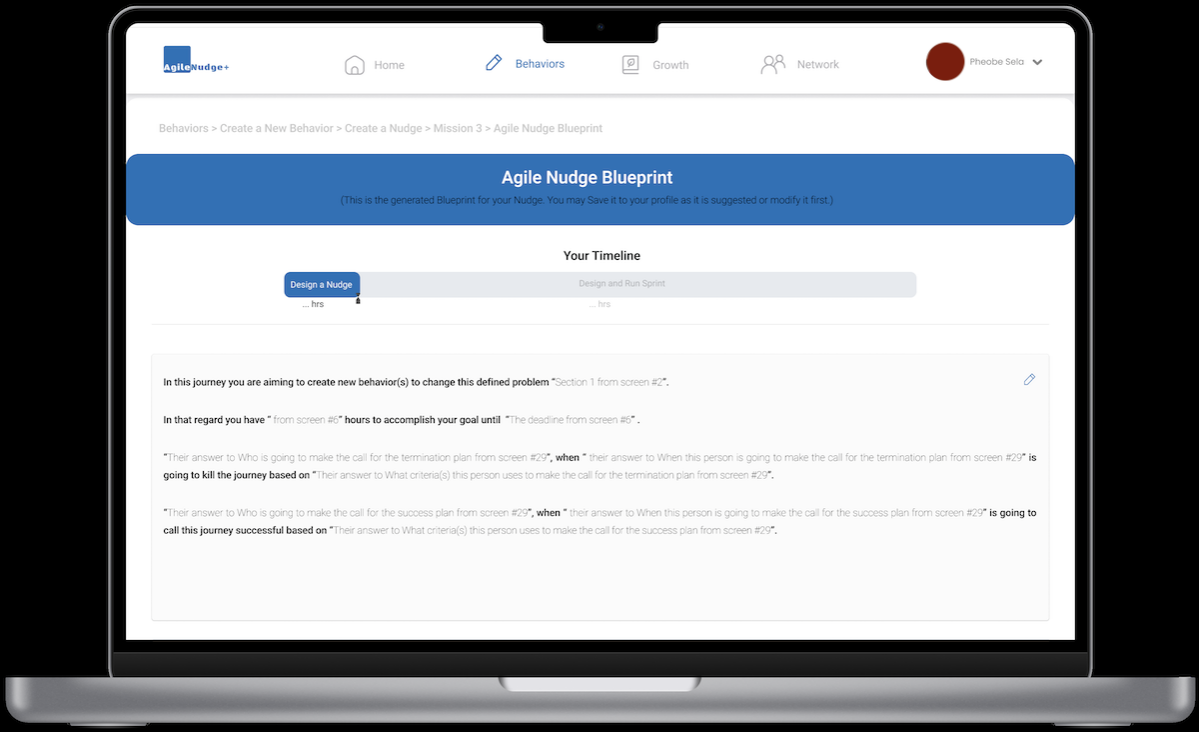
The AgileNudge+ behavioral change journey’s generated nudge blueprint: this screen illustrates the blueprint that users will follow on their way to mapping, designing, and effectively implementing behavioral changes.

**Figure 7:**
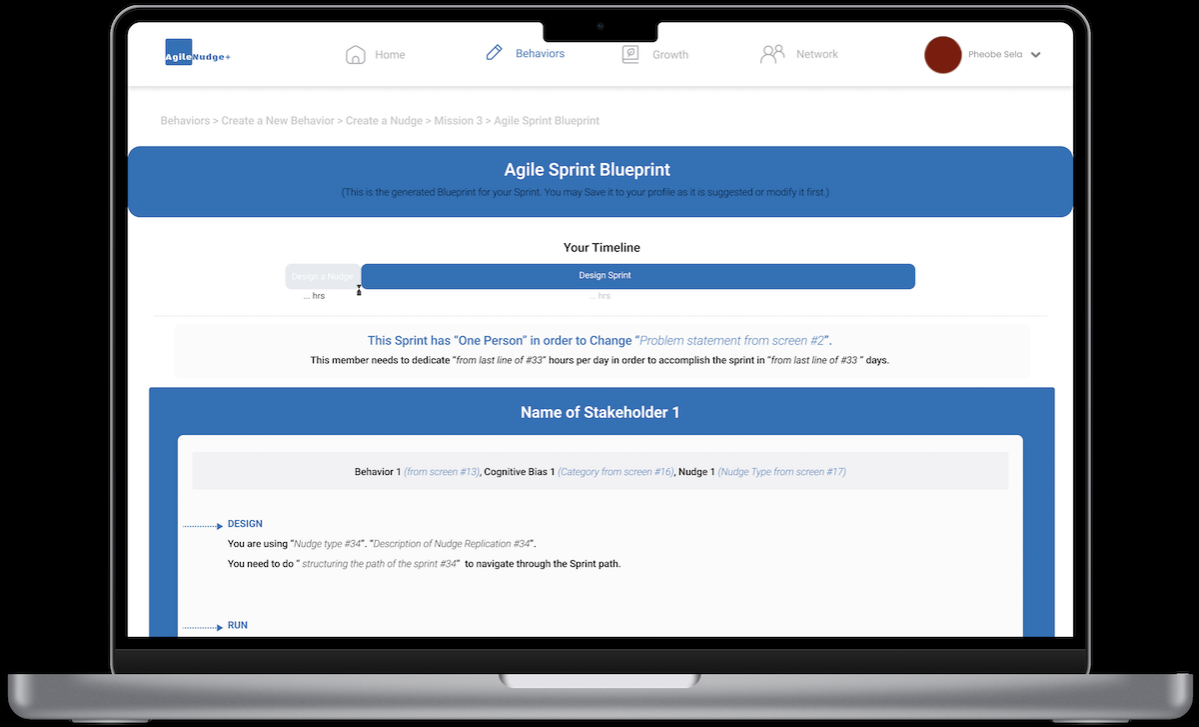
The AgileNudge+ behavioral change journey’s generated sprint bueprint: this screen displays the generated blueprint for the Agile sprint that the users will view. It can be saved to their profile and serves as a reference, helping users stay on track, and understand the steps needed for effective behavioral change.

The AgileNudge+ minimally viable prototype was prototyped using Figma, a web-based collaborative tool for designing interfaces. This platform allowed the design team to observe users’ interactions with the software in real-time (Figures 4-7). The important components and features of AgileNudge+ are described in Multimedia Appendix 3. The transition from a minimally viable prototype to the developed software involved close collaboration between designers and developers. This process was characterized by 7 week-long sprints dedicated to iterative testing cycles of AgileNudge+. Each sprint aimed to refine the minimally viable prototype based on stakeholders’ feedback, ensuring that the software evolved to meet the users’ needs effectively.

### Recruitment

Participants were recruited using convenience and snowball sampling. CHIIS team members facilitated recruitment through digital advertisements by email and word of mouth. Eligibility criteria included minimum knowledge of Agile science, proficiency in English, basic technological knowledge, and access to the internet and Zoom (Zoom Video Communications, Inc) for usability testing and interviews.

### Ethical Considerations

Study approval was obtained from the institutional review board (IRB#23599) at Indiana University, as exempt, before recruiting participants. Informed consent was obtained verbally from all participants before their involvement in the study, and participants were given the option to withdraw at any time without consequences.

Privacy and confidentiality standards were rigorously upheld throughout the study. All data collected were deidentified before analysis, and participants’ identities were not disclosed at any point. Protective measures, including secure data storage and anonymization, were implemented to safeguard participants’ privacy. To maintain the privacy of participants, no images were used in this manuscript that would identify any participants, and previous approval and consent were specifically obtained for all quotes referenced.

No compensation was provided to participants for their involvement in this study as participation was entirely voluntary, and no financial incentives were offered at any time during the study.

### Usability Testing Sessions and Interviews

**Figure 8:**
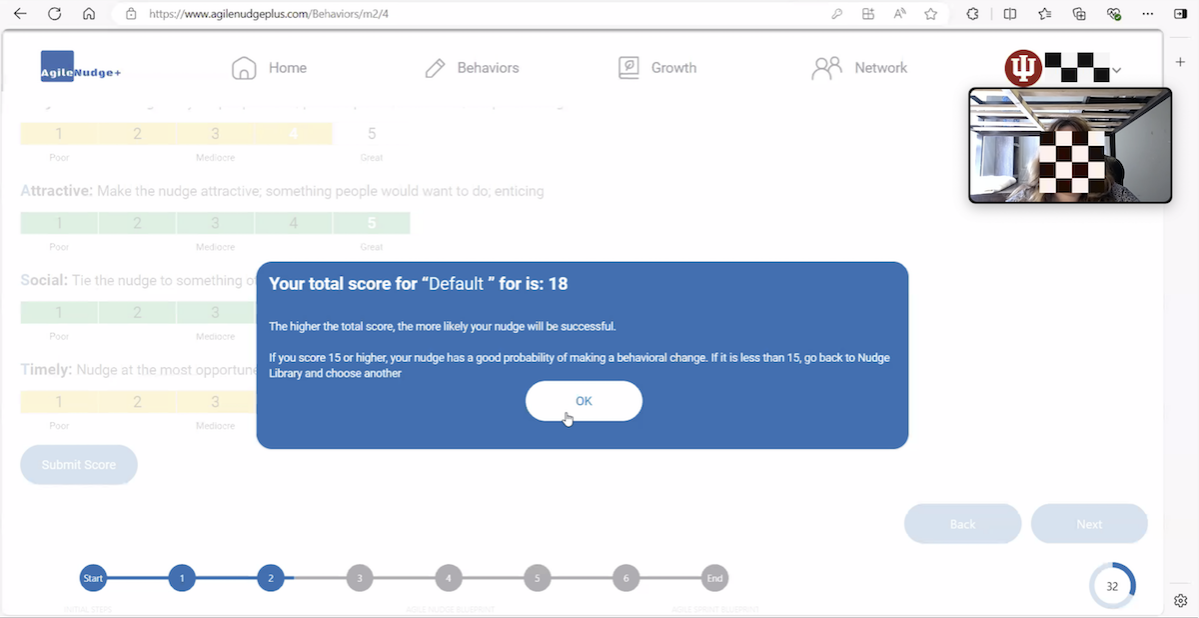
Usability testing session with a user, showing the user working with AgileNudge+ on their computer in a Zoom session.

A total of eighteen 1-hour usability testing sessions for AgileNudge+ were conducted through Zoom, facilitated by 2 design team members (Fereshtehossadat S and Fatemehalsadat S) and 1 development team member. Participants accessed the software through a provided link with no additional installations required and were asked to “Consider a research project of your own, identify a problem, and use AgileNudge+ to plan a behavioral change to address this problem” (Figure 8).

Following each usability test, a semistructured interview was conducted to capture feedback on users’ overall experience, specific challenges, useful and desired features, their self-rated knowledge of Agile science on a scale from 1 (lowest) to 7 (highest), and their rating of AgileNudge+’s usefulness on a scale from 1 (lowest) to 7 (highest).

### Data Analysis

Qualitative data from the usability testing sessions and interviews were analyzed using thematic analysis, following Braun and Clarke’s [32] framework and O’Brien and colleagues’ [33] approach for qualitative research. For the mixed methods study, we adhered to O’Cathain and colleagues’ guidelines [34].

This process involved identifying the frequency of mentions, detecting repeated themes and patterns, and conducting a comparative analysis between the themes and the frequency of challenges versus useful and desired features. This approach provided a comprehensive understanding of user experiences and identified areas for improvement in AgileNudge+.

### Participants

Table 1 summarizes key demographic information about the participants involved in the usability testing sessions and their individual ratings on the software’s usefulness. Participants’ average education level is 17.78 (SD 2.46) years, average experience in Agile science was 2.73 (SD 1.82) years, average knowledge of Agile science was 3.72 (SD 1.18), and average usefulness rating of software was 3.83 (SD 1.67).

**Table 1:** Participant demographics and software evaluation metrics used to assess AgileNudge+ in the usability testing phase.

Participant code	Educational level	Experience in Agile science (years)	Scale of knowledge of Agile science (1-7)	Usefulness of software compared with manual intervention (1-7)
P1	Master	7	5	2
P2	Bachelor	2.5	3	3
P3	MD	1	5	3
P4	Bachelor	4	2	5
P5	Bachelor	6	3	7
P6	Bachelor	Less than 1	3	7
P7	Bachelor	5	4	5
P8	PhD	5	6	3
P9	Bachelor	2.5	5	5
P10	MD	3	5	4
P11	Bachelor	3	3	5
P12	Master	5	2	4
P13	Bachelor	3	4	5
P14	Bachelor	2	3	1
P15	MD, PhD	4	5	2
P16	Master	6	5	6
P17	Bachelor	Less than 1	2	2
P18	Bachelor	2	2	3

## Results

This section presents the findings from the usability test sessions, offering both quantitative and qualitative insights into user experiences with the software.

### Quantitative Results

The initial sample for the usability test consisted of 18 participants. The usefulness of the software was rated on a 7-point Likert scale where 1 was the lowest and 7 was the highest. The mean usefulness rating of the software was 3.83 (SD 1.67). The 95% CI for the mean rating was calculated to be 3.00-4.66.

### Qualitative Results

The qualitative analysis of usability test sessions revealed 3 key themes within user feedback: the demand for offering guidance content, the need for time-efficient interactions with the software, and the impact of integrating gamification within technological activities.

#### The Demand for Offering Real-Time Guidance Content

The data analysis indicates that AgileNudge+ facilitated participants’ completion of the nudge intervention design process. Participants found that the software effectively organized the necessary steps to design a nudge intervention within a single platform. For a detailed example of this process, refer to Textbox 1, which illustrates P11’s nudge design aimed at reframing recruitment mindset and meeting targets.

The systematic approach of AgileNudge+, from the participant’s point of view, encouraged them to engage deeply with their research problem, leading to more precise problem statements and better nudge choices and sprint planning in addition to preventing them from skipping crucial steps.

Oh, this is so interesting...stuff we naturally do; it’s interesting to think of it!

P12, master’s degree

For instance, the initial steps of AgileNudge+ particularly prompted users to thoroughly consider their research problem and the ideal behavior they sought to influence. Participants claimed that this detailed questioning not only helped in clarifying the problem but also in identifying the actual demand for a nudge. In some cases, the software indicated that there was no demand to proceed with nudge design based on the input data. This feedback led participants to refine their problem statements, ensuring that they addressed the most accurate issue within their project. Users appreciated this feature, as it helped them avoid spending time on irrelevant problems and encouraged deeper reflection on their project’s core issues.

These questions are very good...These questions are very, very good. I really like them.

P16, master’s degree

In addition, participants valued the software’s provision of overviews and descriptions for each step, which guided them through the nudge design process. However, some users, particularly those with less experience in Agile science, found the high-level terminology challenging. They expressed a need for more guidance to understand these terms better during task completion.

**P11’s example of nudge to reframe recruitment mindset and meet targets.**

Participant 11 (P11), a clinical trial research specialist, struggled to meet recruitment targets while facing challenges with staff turnover and burnout. P11 recognized the need to shift the recruitment team’s mindset to achieve better outcomes, especially since they lacked timely feedback and accountability. To address these challenges, P11 used AgileNudge+ to reframe recruitment staff members’ behaviors, focusing on improving staff engagement and effectiveness in reaching potential participants.

Through the software’s cognitive bias tool, P11 identified key biases affecting recruitment staff and implemented a nudge strategy centered on ambiguity, priming, salience, and affect biases. The designed nudge aimed to reframe how recruitment staff approached their interactions with potential participants by modifying recruitment scripts and dashboards. Specifically, a preliminary screening question (priming), emphasis on the study’s commitment and impact (salience), and emotion-focused phrases (affect) were to be incorporated into the script, while visual prompts for patient follow-up (ambiguity) or staff engagement (affect) were designed to be embedded in the spreadsheet where recruitment data were tracked. After 2 iterations refining the nudge using EAST and MINDSPACE frameworks, P11 designed a team sprint to track progress and enhance accountability. They created a scalable framework for long-term success, with plans for frequent evaluations and a clear success and termination plan for the designed nudge (a step-by-step account of the process is described in Multimedia Appendix 4).

#### The Need for Time-Efficient Interactions With the Software

Participants in our study found AgileNudge+ useful, particularly appreciating the integration of additional resources such as CHIIS-developed Cognitive Biases Library and the Nudge Library. These resources were invaluable for users who were not highly familiar with these concepts or who had limited knowledge in Agile science. Participants stated that the availability of these resources within the software helped them accurately identify cognitive biases and nudge types, ensuring they did not overlook any critical information and saving time that would otherwise be spent searching for external resources. However, participants did find navigating through the extensive list of information to be challenging and time-consuming. Specifically, they noted that the time required to plan a behavior change (approximately 1 hour) was excessive and mostly spent going through the libraries. Despite their necessity, this frustration led some users to either ignore the resources or select options hastily to proceed quickly. Users suggested simplifying this process or automating information retrieval to enhance efficiency.

There are so many [cognitive biases in Cognitive Bias library]. I’m just going to choose anyone right now...I am just going to leave it there!

P10, MD

While the detailed substeps of the behavioral change process were thorough, users found it time-consuming to navigate the list of questions. Although this comprehensive approach ensured all aspects were considered, some participants stated that it detracted from their ability to focus on the overall process. Participants emphasized the need for a more time-efficient process to maintain their focus and productivity.

#### The Impact of Integrating Gamification Within Technological Activities

The integration of gamification elements with technological activities within the AgileNudge+ software had an impact on our users’ experiences. The software included a reward system based on accumulating points as users progressed through steps. During the semistructured interviews, users noted that while they found the reward system motivating, it was not entirely clear during task completion. They struggled to understand the points earned after each mission, mainly due to challenges with the user interface and the location of the reward system element on the screen. Despite these challenges, users found motivation in the reward system, considering it as a driving force during missions that helped them emotionally navigate easier through different steps in the software.

The ones where the score was, definitely [motivating]. I felt confident that I was at a good place with it. And then the one that I scored below...it got me to understand...what we’re behind in, and what I maybe should focus in!

D4, bachelor’s degree

In addition, AgileNudge+ software featured a progress bar at the bottom of each screen to indicate the user’s progress during missions. Data from our study revealed that the progress bar served as additional motivation, offering users a visual representation of their behavioral change journey. This visual feedback, according to our participants, not only encouraged users to continue their project but also provided a sense of accomplishment as they advanced through the tasks.

## Discussion

### Principal Findings

The findings of our study highlighted the importance of providing timely guidance within the software and ensuring time-efficient interactions between the user and AgileNudge+. We discovered that AgileNudge+ is effective for modifying behaviors within health care research settings by helping users think deeply. The steps provided helped users identify the problems in their research project and the interface enabled users to design and test nudge interventions through sprints. Furthermore, the development of AgileNudge+ highlights the feasibility and benefit of incorporating technology into the innovation and implementation of evidence-based health care interventions. AgileNudge+ increases the efficiency of nudge creation and implementation while being accessible to users who are not experts in Agile science and are located at any English-speaking institution. After analyzing data from our usability testing sessions and user interviews, we identified two main areas for improvement: (1) maintaining an atomized process and (2) enhancing user motivation through gamification mechanisms. Taking participant feedback and previous scholarly works into consideration, the second version of AgileNudge+ software will be redesigned based on these themes.

#### Maintaining an Atomized Process

AgileNudge+ is designed to facilitate behavior change within complex adaptive human organizations, using frameworks rooted in Agile science [3-5,35]. The software’s design process followed Agile science principles, such as iterative design and sprint cycles, and its content builds on decades of work in implementation science to optimize structured nudge design, addressing past limitations in education time, finances, and accessibility [10,20,21]. Our pilot study, with 18 users, demonstrated that AgileNudge+ positively impacts behavioral change processes by providing users with a structured methodology based on Agile science on a single platform.

Despite the benefits of Agile science, previous attempts to develop Agile software have been limited. The only notable effort was at a Russian university for military purposes, which failed to incorporate nudge theory [36]. Other project management strategies like Lean and Six Sigma have previously been developed into software to increase the efficiency of health care innovations [37-39]. However, these programs do not specifically focus on the creation, implementation, and diffusion of evidence-based nudges. In contrast, AgileNudge+ was specifically designed based on Agile science principles to address this gap. We hope that by providing full transparency of the Agile design process used to create AgileNudge+ will aid further development of implementation-driven software.

While digital nudges have been developed within the health care context, no program has yet used technology to facilitate the development of nudges [40-42]. By breaking down each step of the Agile implementation and innovation processes into a series of structured questions, AgileNudge+ helps users navigate complex steps within a project management context. The benefits of this are exemplified by participants contending that the software helped them think deeper about their problems and see hidden aspects of the nudge design process.

AgileNudge+ is uniquely designed for health care delivery systems, incorporates nudges and sprints, and allows users to design their nudges by accessing the software’s cognitive bias and nudge libraries. Agile science distinguishes itself from other strategies by accounting for interpersonal cognitive biases and considering the broader network into which a solution is introduced [10,43]. AgileNudge+ can be used to streamline the development of a diverse array of nudges, from text reminders that improve study enrollment to storytelling that creates demand for new health care models within the delivery system [44,45]. Thus, we believe that to date, AgileNudge+ is novel in its integration of structured Agile project management strategies and its focus is on maximizing the efficiency, ease, and success of nudge-related health care solutions.

Given the vast breadth of information held in the cognitive bias and nudge libraries, participants noted feeling overwhelmed when attempting to navigate through these resources, despite their importance. Based on user feedback and technological possibilities, we plan to address these issues by incorporating artificial intelligence (AI) into the next iteration of AgileNudge+. AI is a contemporary technology applied across various fields, including health care [46]. It has demonstrated its importance through studies on explainable AI and its potential to support health care providers in decision-making [47]. By analyzing external data and performing tasks traditionally managed by humans, AI transforms societal functions and impacts behavior, cognition, and lifestyle [47]. By incorporating AI, we will eliminate the step of manually searching the cognitive biases library and nudge library. In the next version, the trained AI will receive the problem from the user and suggest nudge types while allowing the user to modify them as needed. Users can also delete or add any nudge types they want by searching through the categorized libraries provided by AI. AI’s ability to process large volumes of data and uncover previously overlooked correlations has shown promising results in health care, enhancing both research and clinical practice [47,48]. Incorporating AI not only reduces the time spent using the software and increases efficiency but also simplifies the process and helps users reduce their mental workload. Incorporation of AI elements will be streamlined using Agile design processes, focusing on sprinting and implementation to produce a minimally viable updated prototype in an efficient and user-specific manner.

#### Enhancing User Motivation Through Gamification Mechanisms

Gamification, rooted in motivational psychology and game mechanics, integrates video game elements into nongaming systems, driving apps across various domains including productivity, finance, health, sustainability, news, user-generated content, and tutorials [49,50]. By incorporating elements such as reward systems, point scores, badges, levels, and leaderboards, gamification aims to make interactions more engaging [51].

In AgileNudge+, the gamification strategy includes a point system to boost user engagement by rewarding interactions with the software. The point system and tracking system serve as nudges themselves, encouraging users to continue using the software. In the first version, the goal was to reach 100 points, whereas in the second version, the goal was to change the project status to “Success.” The positive impact of gamification extends to emotion, fostering motivation, enthusiasm, enjoyment, satisfaction, interest, and innovation [52]. Participants in our study were gently nudged toward task completion and goal achievement, enhancing the overall user experience and positioning AgileNudge+ as a more applicable tool through the incorporation of gamification.

Our data analysis showed that this approach effectively motivated participants, encouraging their interaction with the software. However, given the nature of the tasks (ie, solving a problem within a research project by changing related behaviors), users could bypass the software and complete the task manually. Consequently, we determined that although the reward system was motivating, given the variety of gamification options, AgileNudge+ could benefit from more gamified motivation elements. Therefore, based on the user’s habits in the behavioral change process, AgileNudge+ will focus on tracking progress rather than rewarding task completion in the next iteration. To simplify the steps and maintain motivation, we will incorporate AI assistance and a tracking system. AgileNudge+ will prompt users to update their progress periodically (daily, weekly, or monthly) as they implement nudges through sprints and the status of their projects will be displayed using color codes (green indicating success, yellow indicating in tolerance, and red indicating terminate) based on provided deadlines.

Grounded in self-determination theory [49,53,54], previous studies have shown that gamification aligns intrinsic motivation, extrinsic motivation, and motivation with fundamental human needs—competence, autonomy, and relatedness—creating motivating experiences that maintain desired behaviors in health care [55,56]. Given that gamification enhances motivation and performance in technology-enhanced learning [54] and considering that AgileNudge+ requires users to undertake diverse and sometimes lengthy steps reflecting Agile science principles, we believe users need both internal and external motivation to maintain their interest during interactions with the software.

Studies have shown that digital behavioral data can offer higher temporal resolution compared with traditional behavioral experiments [57]. Incorporating gamification into AgileNudge+, a tool that supports this data-driven approach, enhances its effectiveness. The finer temporal resolution of digital data helps the tracking system in AgileNudge+, allowing users to stay motivated and monitor their progress more accurately. This enables them to adjust their strategies promptly, improving the overall impact of the tool.

The initial version of AgileNudge+ included gamification elements, these elements will be revised for the second version to better align with data from usability test sessions and interviews. Our data indicated that due to the importance of nudge implementation, users would design nudge interventions regardless. However, the tracking system will help them stay motivated to monitor their progress and promptly terminate a sprint if the data indicate that the chosen nudge is unsuitable, thereby encouraging them to select a more effective one. This level of accuracy in tracking and decision-making would be difficult to achieve without using the software. Therefore, gamification enhances their ability to improve accuracy and effectiveness.

### Limitations and Future Study

Our study offers valuable insights into the integration of technology for applying Agile science in fostering positive behavioral change; nonetheless, there remain several limitations. One of the primary limitations of this study is the small sample size of 18 health care professionals and researchers. While sufficient for initial usability testing and a mixed methods analysis, this limited sample restricts the generalizability of our findings and our understanding of how a larger cohort of users might view the software. The goal of the AgileNudge+ software is for it to be accessible and beneficial to a geographically diverse range of health care professionals and scholars to maximize the number of positive health care interventions. In addition, convenience and snowball sampling methods have the possibility to introduce selection bias and may not fully represent the broader population of health care professionals and researchers. A larger and more diverse sample would provide more comprehensive insights into the usefulness of AgileNudge+. The usability testing was conducted through Zoom, which may not reflect real-world usage of AgileNudge+. The remote, 1-hour sessions might also have influenced user behavior and feedback, not capturing the extended, iterative nature of using the software in long-term projects. Further research should focus on longitudinal studies that observe users as they integrate AgileNudge+ into their regular workflow and project management processes, providing a more accurate assessment of its impact and effectiveness. Finally, the study relied on self-reported measures for assessing participants’ knowledge of Agile science and their rating of AgileNudge+’s usefulness. While convenient, self-reported data can potentially overestimate or underestimate a participant’s abilities and perceptions. To address this, future research should compare the outcomes and effectiveness of AgileNudge+ against results from without the software to provide a more objective evaluation of its impact.

### Conclusions

In summary, AgileNudge+ is a novel step forward in promoting behavioral change using an Agile framework. Through its user-friendly features, the software systematically guides users to create, implement, and test nudges, enhancing both the effectiveness and engagement of the behavioral change journey. As a result, AgileNudge+ serves as a valuable and accessible hub that simplifies and improves the nudge creation process to facilitate meaningful, widespread, and evidence-based change within the health care system.

## Appendix

Multimedia Appendix 1 Agile nudge cycle guideline.

Multimedia Appendix 2 Checklists to evaluate a nudge.

Multimedia Appendix 3 AgileNudge+ components and features.

Multimedia Appendix 4 Example of nudge design for a recruitment mindset intervention.

## Abbreviations

AI: artificial intelligence
CHIIS: Center for Health Innovation and Implementation Science
MoSCoW: Must Have, Should Have, Could Have, and Won’t Have This Time

## Data Availability

All data generated for this study is included in this published article. Semistructured interview transcriptions are anonymized and restricted for confidentiality. Any further inquiries can be directed to the corresponding author.
