# Supplementary material for: The Feasibility of AgileNudge+ Software to Facilitate Positive Behavioral Change: Mixed Methods Design
Source: JMIR Form Res. 2024 Nov 13;8:e57390. doi: 10.2196/57390 (PMC11602761; doi:10.2196/57390)
Supplement: Multimedia Appendix 1 [file formative_v8i1e57390_app1.doc]

## **Multimedia Appendix 1: Agile Nudge Cycle Guideline**

### **What is a Nudge?**

A nudge is a behavioral intervention that that architects the social, physical, or digital environment to facilitate a certain behavior without forbidding choice.

### **What is the Agile Cycle?**

The agile cycle is a project management strategy that is based on demand. Demand facilitates whether or not there is a need for an evidence-based solution. The agile cycle is composed of thinking and doing. The thinking process includes planning, reflecting, and adjusting, and only 10% of time should be allocated to this. The doing process consists of sprints, or iterative testing cycles that are continuously revised based on actionable, timely, nonjudgmental feedback, which should occupy 90% of the allocated time. Higher demand means that the agile cycle can occur quickly. With low demand the cycle is slow-moving and can get stuck.


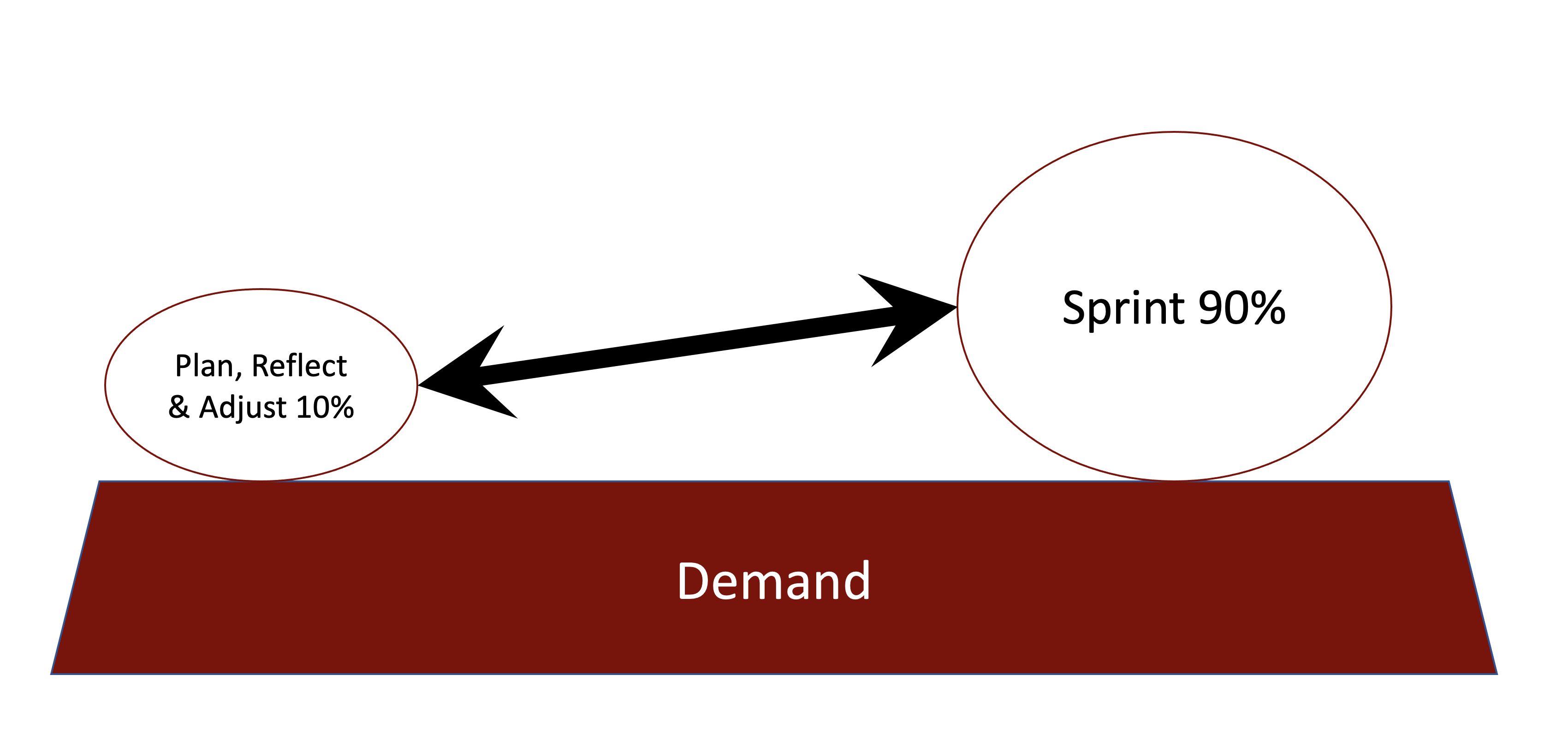
The Agile Cycle.

### **What is Agile Implementation?**

Agile implementation is an 8-step process. The steps of agile implementation include identifying opportunities, identifying evidence-based healthcare services, developing evaluation and termination plans, assembling a team to develop a minimally viable service, performing implementation sprints, monitoring implementation performance, monitoring whole system performance, and developing a minimally standardized operating procedure (MSOP). Steps 1-4 are planning steps and steps 5-8 are execution steps.

#### **Step 1: Identify Opportunities for Systematic Improvement**

The individual looking to create change must work with organizational leaders, community leaders, and clinical providers to identify opportunities for implementing evidence-based healthcare services. After identifying an opportunity for change, demand must be confirmed, with organizational investment of time, financial, and social capital into the project. The demand for the project must be assessed at every subsequent step of the process.

***Step 2: Assess Evidence-Based Healthcare Services***

Systematic evidence reviews must be used to identify evidence-based practices that address the clinical opportunity identified. The selected evidence-based practice must deliver the quadruple aim of better care, better health, lower cost, and improved patient/clinician experiences.

***Step 3: Develop Evaluation and Termination Plans***

An evaluation protocol must be developed for the selected evidence-based practice. The evaluation plan should include organizationally appropriate measures, service type, implementation goals, as well as scheduled milestones and quality indicators of success (can be quantitative or qualitative). The evaluation plan should emphasize the criteria for de-implementing the evidence-based solution as early as possible if it is considered a failure. The termination plan should include the de-implementation timeframe and who is responsible for making the decision to ultimately stop the implementation process.

***Step 4: Assemble a Team to Develop a Minimally Viable Service***

A local and diverse implementation team must be built to convert the selected evidence-based practice into a minimally viable service by localizing the content, delivery process, and expected outcomes of the service. The localization process aims to accommodate the unique characteristics of the local healthcare delivery system and the individuals within it. The minimally viable service is considered the pilot version that is to be implemented and tested in incremental, subsequent iterations.

***Step 5: Perform Implementation Sprints***

Facilitation of self-contained sprint cycles assess as early as possible whether the evidence-based healthcare service is properly localized and evaluates the success of localization. Sprints are a fixed period of focused work performed by designated individuals, resulting in lessons learned that feed into frequent, subsequent redesign-and-sprint interactions. Each sprint requires time, space, and resources, but allows early insight into successes, failures, and opportunities for modifications prior to starting the next sprint.

***Step 6: Monitor Implementation Process***

Care teams must develop performance feedback loops to monitor the fidelity of the selected evidence-based healthcare service. The team must reflect upon what they are learning, gauge impact while acknowledging any conflicts, detect any emerging behaviors, and identify solutions to problems, all the while adjusting the implementation process to maximize fit.

***Step 7: Monitor the Entire Performance of the System***

Create a monitoring system that evaluates the evidence-based service’s impact on the organization's quality, financials, and employee satisfaction results. The purpose of whole-system monitoring is to detect as early as possible any unintended or adverse consequences of implementing the evidence-based healthcare services.

***Step 8: Develop a Minimally Standardized Operating Procedure***

If the implementation of the evidence-based healthcare service was evaluated and found to be meeting internal demands and goals, the team should develop a minimally standardized operating procedure manual. The manual is updated on a regular basis and should help spread the successful evidence-based healthcare service across other practices or communities.


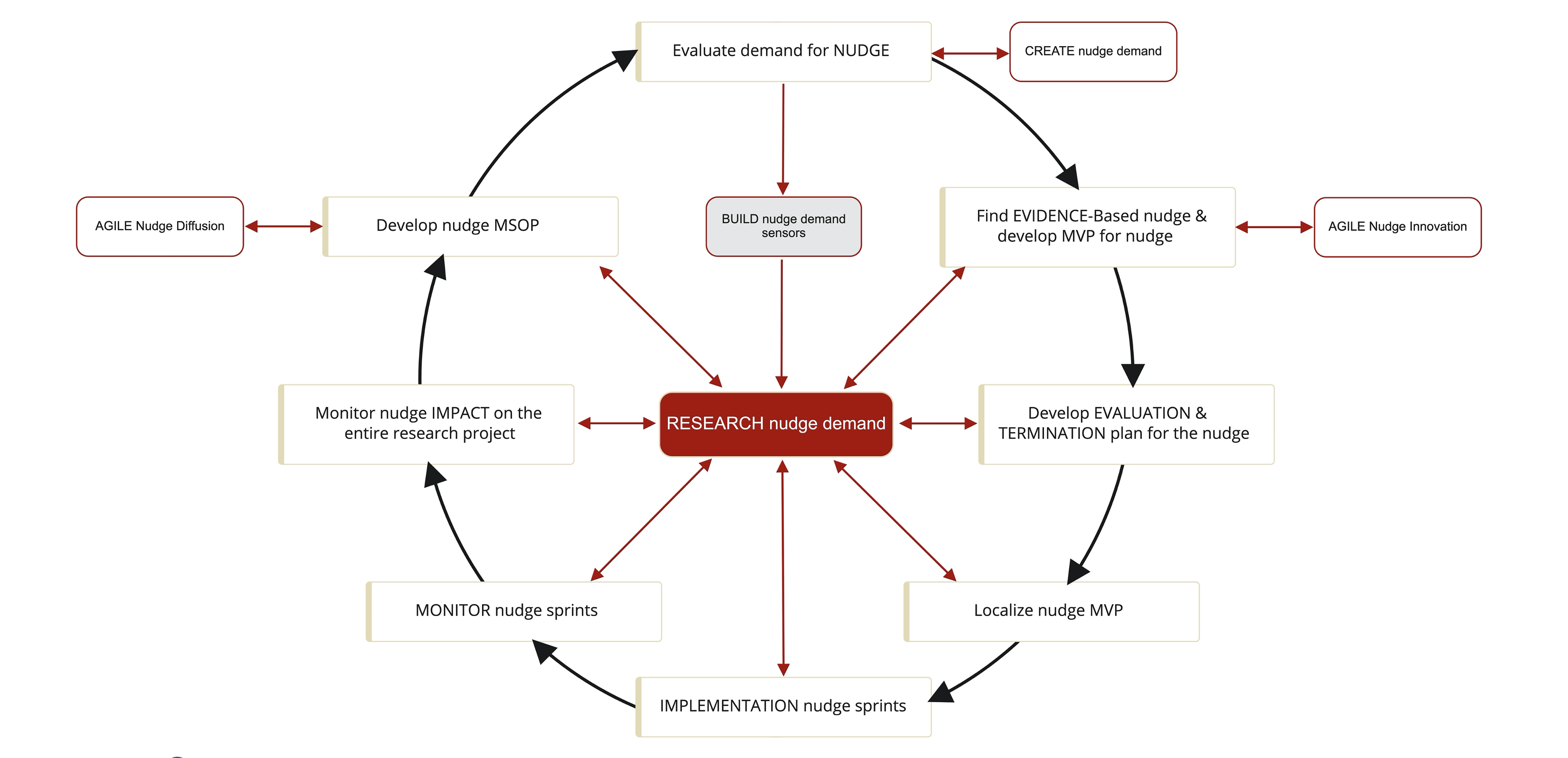
Agile Implementation Cycle.

### **What is Agile Innovation?**

Agile innovation is an 8-step process. The steps of agile innovation include confirming demand, studying the problem, scanning for solutions, creating evaluation and termination plans, ideating and selecting a solution, doing innovation sprints, validating solutions, and packaging for launch. Steps 1-4 are planning steps and steps 5-8 are execution steps.

***Step 1: Confirm Demand***

This involves verifying support and assess demand for the nudge.

***Step 2: Study the Problem***

One must investigate the current state to identify the needs of the organization.

***Step 3: Scan for Solutions***

By using systematic evidence-reviews, this requires scouting and analyzing existing solutions or processes for the target problem.

***Step 4: Plan Evaluation and Termination Plan***

Similar to the implementation process, structured processes are required to determine when to proceed with a solution or when to stop and reflect a targeted innovation solution.

***Step 5: Ideate and Select***

Members must collect ideas and select top candidates for sustainable solutions.

***Step 6: Do Innovation Sprints***

Sprints alternate between iterative testing cycles and adjusting the prototype as needed.

***Step 7: Validate Solutions***

After running sprints, a final analysis must be conducted on the selected solution with an emphasis on evaluating any potential unexpected consequences.

***Step 8: Package for Launch***

This step requires creating a business plan for the minimally viable product selected.


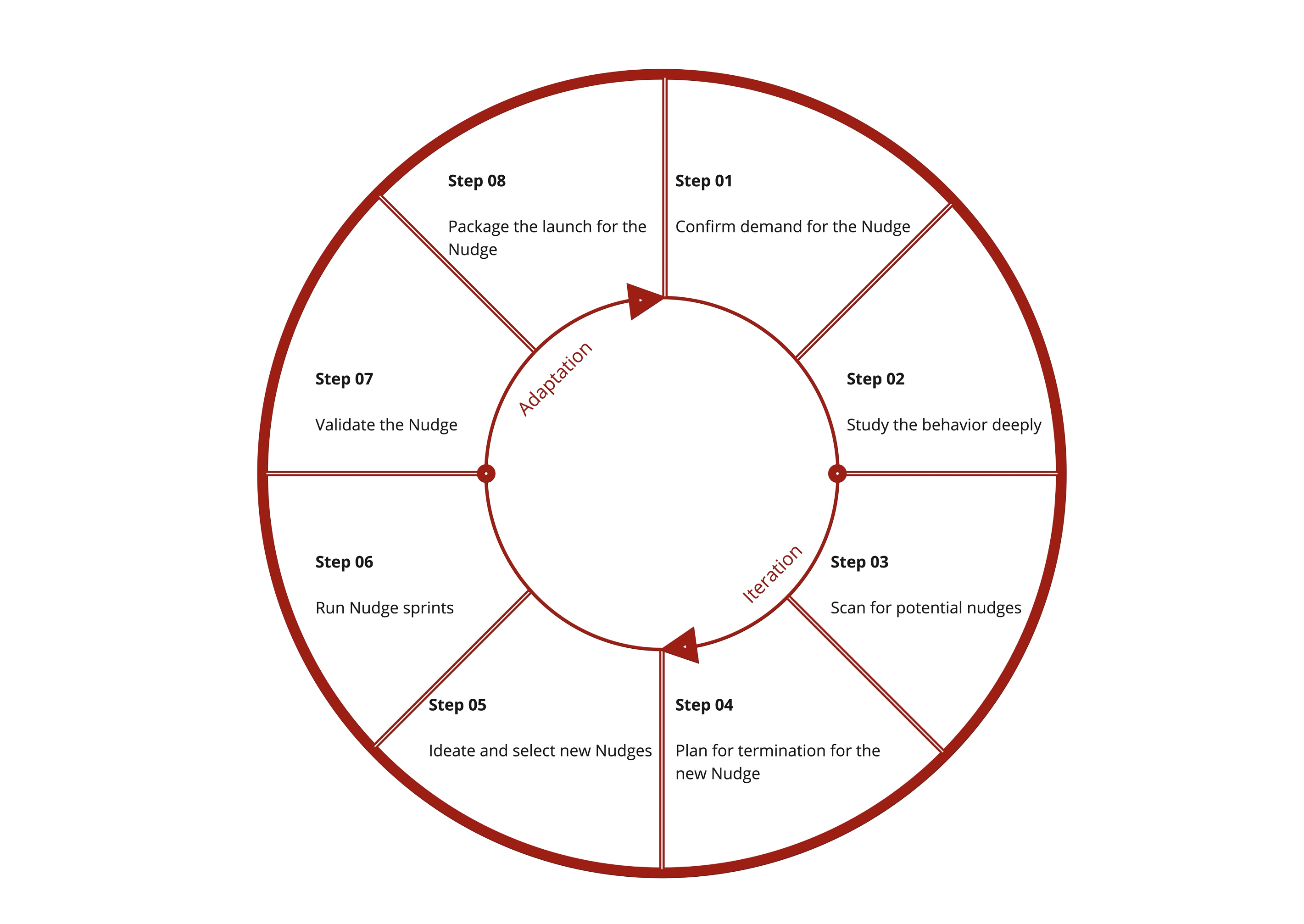
Agile Innovation Cycle.

**What is an Agile Sprint?**

An agile sprint is allocated time and space to foster collaboration and innovation between peers. Sprinting is a way to properly test a minimally viable product that is based on iterative testing cycles and immediate feedback. Weekly huddles are required to allow a team to collectively plan, reflect, and adjust the sprint as needed. The two types of sprints are team sprints and relay sprints. In a team sprint every member is present at the same time in a meeting, working together to plan the sprint, reflect on a sprint cycle, and update the product before conducting the next sprint cycle. A relay sprint is where one member picks up where another left off, allocating specific steps of a sprint process to certain individuals matched with their specific skill-sets to complete sprint cycles in an asynchronous and chronological format.

**Designing a New Nudge...**

The first section of the agile nudge cycle tool will have a page that designates the title of the sprint related to the nudge, the nudge label, who the point person is, and what date the sprint will occur on. At the bottom of the screen the tool will ask for the objective of designated nudge.

1. Confirm demand using quantitative data that backs up a clear, concise problem statement. Introduce a stakeholder agreement signed with the date.
2. Map the digital, physical, and social environment surrounding people who are targeted for behavioral changes by answering four questions:

- Who is the person targeted, and what is the current behavior that needs to be changed?
- Who are the people interacting with the targeted person? Do they have any current behavior that is contributing to the target person’s current behavior?
- Is there any existing messenger that could be used as a nudge carrier or a nudge?
- Are there any existing digital, physical, and social artifacts (or nudges) that are contributing to the current behavior of the targeted person?

1. Map the behaviors using the recruitment and retention journey: stranger to acquaintance to engager to interested to consent to completer to fan. Between each section exists the potential for a nudge. Specify the targeted behavior of interest within the journey with evidence to justify the nudge, providing an effect size if the evidence has a similar population to the current project.
2. Search the cognitive bias library to identify the cognitive bias that might be leveraged to design a Minimally Viable Nudge to accomplish the target behavior. Take into account that humans have faulty memories, need to act fast, are overwhelmed if there is too much information, and look for meaning.
3. Select the milieu of the nudge (physical, digital, social) and use the MINDSPACE checklist to select the category for designing the Minimally Viable Nudge and then check the compatibility of the Minimally Viable Nudge using the EAST checklist (see Appendix 2).

- The EAST checklist is a way to gauge the potential success of your new nudge prior to testing the new nudge in a series of real-world sprints. The goal is to make your nudge Easy, Attractive, Social, and Timely. For each of these categories the nudge is rated 1 (poor), 2, 3 (mediocre), 4, or 5 (great). The scores are summed and a score of 15 or higher indicates that the nudge has a good probability of making a behavioral change.
- The MINDSPACE checklist is a quick way to know if your nudge is effective enough to use in a real-world setting. The higher the nudge score, the more effective the nudge. The categories are messenger, incentives, norms, defaults, salience, priming, affect, commitments, and ego. Similar to the EAST checklist, each item is scored 1 (poor) - 5 (great) and scores are summed. If the score is 35 or higher, it is capable of affecting behavioral change.

1. Define the Termination Plan for both the selected Minimally Viable Nudge as well as a termination plan to stop working on the targeted behavior. State the goal, the evidence-based nudge and why it is being used, a description of how one is pulling data, where and how often, an acceptance benchmark, and a kill benchmark explaining who, when, and what is required to kill the nudge.
2. Run a series of sprints to test the Minimally Viable Nudge. Specify what the task for the sprint is. If the task requires one member, specify how many hours the member needs to work, then multiply that number by 2 and use the value to decide the length of task completion per day and the number of days. If multiple members are needed, define the minimally viable members, decide if they need to work simultaneously or not, decide what each member will contribute, how many hours they will spend, and how many days they will need. Then choose to run a team or relay sprint. In any sprint that is run, describe the minimally viable nudge, specify the length of the sprint, the start date, the end date, the evaluation date, the site of data collection, the frequency of data evaluation, the goal, and the results. Do this as many times as are needed.
3. Finally, describe a minimally standardized operating procedure to establish the nudge that was maximally successful and describe how the nudge will be scaled and standardized.
