# Supplementary material for: The Feasibility of AgileNudge+ Software to Facilitate Positive Behavioral Change: Mixed Methods Design
Source: JMIR Form Res. 2024 Nov 13;8:e57390. doi: 10.2196/57390 (PMC11602761; doi:10.2196/57390)
Supplement: Multimedia Appendix 2 [file formative_v8i1e57390_app2.doc]

## **Multimedia Appendix 2: Checklists to Evaluate a Nudge**

### **MINDSPACE Checklist**


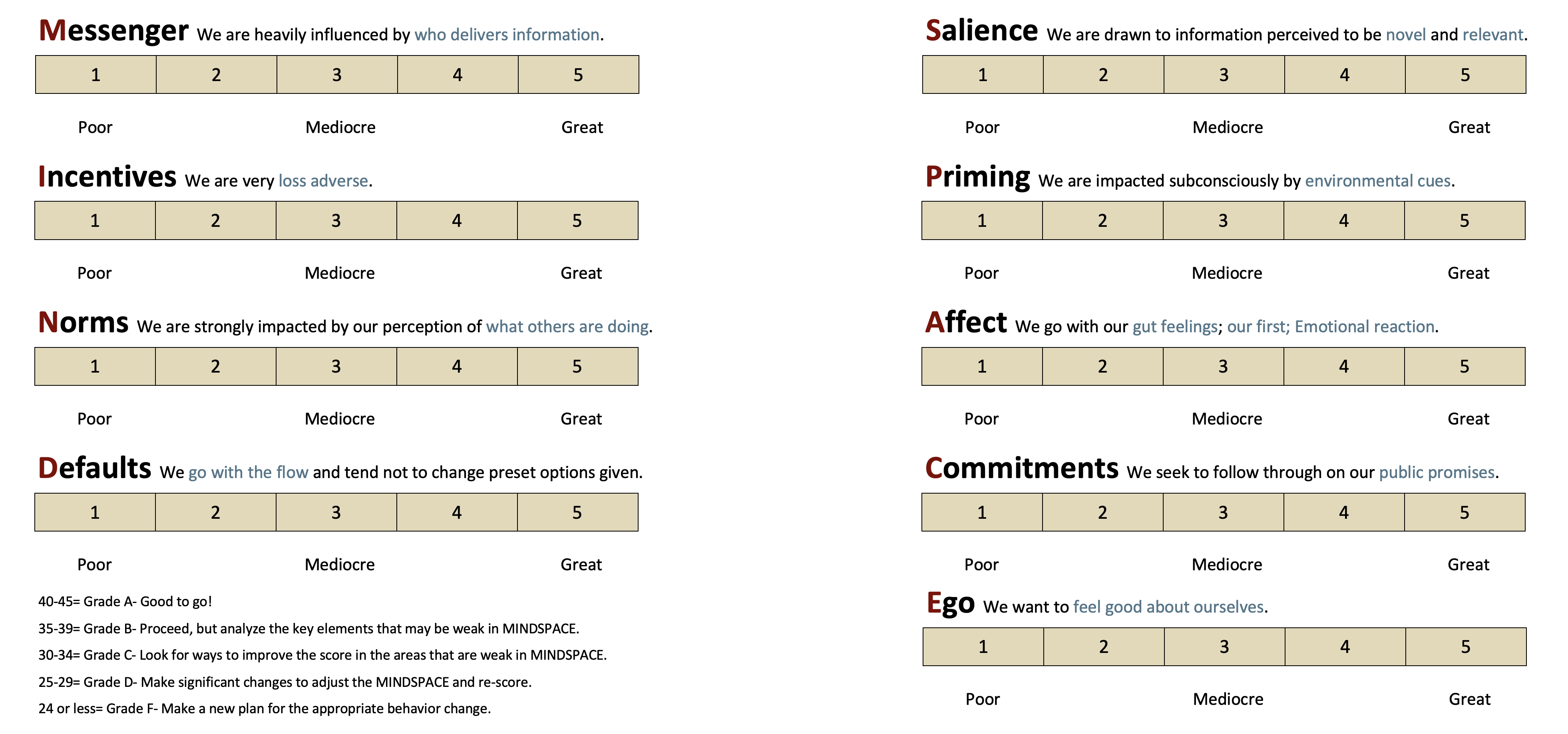
MINDSPACE Checklist.

### **EAST Checklist**


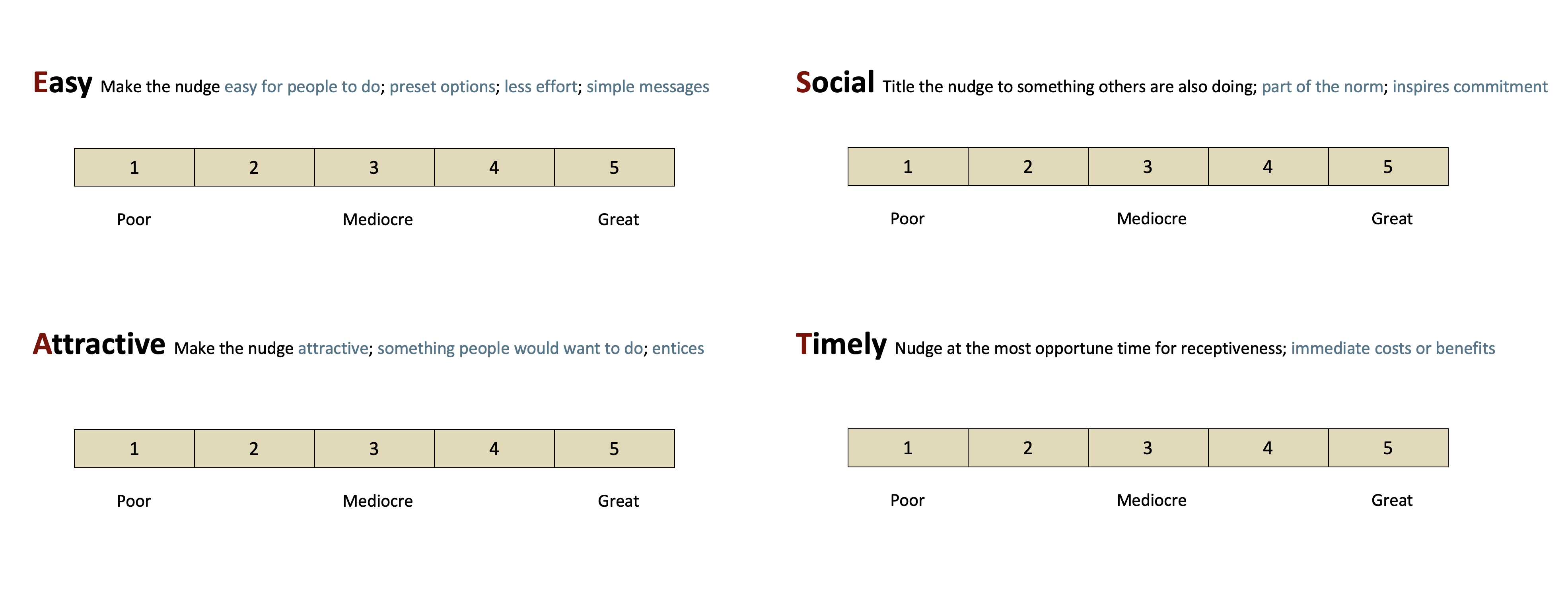
EAST Checklist.
