# Supplementary material for: The Feasibility of AgileNudge+ Software to Facilitate Positive Behavioral Change: Mixed Methods Design
Source: JMIR Form Res. 2024 Nov 13;8:e57390. doi: 10.2196/57390 (PMC11602761; doi:10.2196/57390)
Supplement: Multimedia Appendix 3 [file formative_v8i1e57390_app3.doc]

## **Multimedia Appendix 3: AgileNudge+ Components and Features**

*Homepage:* Upon signing in or signing up for their initial use of AgileNudge+, users will be seamlessly directed to the homepage. Here, they will encounter the three primary modules of the software: Behaviors, Growth, and Network. Additionally, users will be provided with a preview of significant news under the "Highlights" section, offering a concise overview of noteworthy updates and information within CHIIS (Figure 4).

### **Behaviors Section**

In the "Behaviors" section, users start a transformative journey within the software, driven by a two-step process: Nudge and Sprint. Serving as the central module of the application, this section facilitates the documentation of current behavior, ideal behavior, and essential details crucial for behavioral change. Structured around key stages, users navigate through a series of carefully crafted steps:

***Behavioral Snapshot***

Users capture a comprehensive overview of their existing behaviors, laying the foundation for subsequent stages while recognizing and defining key stakeholders and influential messengers who may impact the user's behavior and could shape and support the behavioral change journey.

***Nudge Crafting***

Utilizing the cognitive bias library and nudge library to design personalized nudges aligned with behavioral goals.

***Effectiveness Evaluation***

Assessment of nudge impact through the EAST and MINDSPACE checklist, accompanied by personalized scores and messages (Appendix 2).

***Planning for Success***

Development of comprehensive termination and success plans, charting the course for sustained behavioral change.

Furthermore, the section provides guidance for designing and executing sprints, allowing users to decide between individual or group participation. Users can monitor sprint progress by providing thoughtful responses to targeted questions. Nudge blueprints and sprint blueprints are seamlessly integrated, offering structured frameworks to support users throughout their transformative journey.

***Mapping the behavioral change journey***

From the outset of the behavioral change journey, AgileNudge+ software offers a visual map designed to streamline the user's tracking of various components: current behavior, stakeholders, cognitive biases, associated nudges, and the desired ideal behavior, along with their interconnections. This comprehensive overview map becomes accessible to the user after the completion of each mission, serving as a visual aid to recall elements and their correlations whenever necessary (Figure 5).

***Nudge Blueprint***

The first three missions in the Behaviors section are focused on formulating the appropriate nudge(s). Upon the successful completion of these first three missions, AgileNudge+ offers a "Nudge blueprint" – a concise summary highlighting the most crucial questions posed and the corresponding user-provided answers. This Nudge blueprint is conveniently available for users to save, share, or print, serving as a valuable guide for future reference (Figure 6).

***Sprint Blueprint***

In the final three missions of the behavioral change journey, the last three missions aid users in designing, monitoring, and reflecting on the sprint. Following the completion of these missions, AgileNudge+ furnishes a "Sprint blueprint" – a comprehensive summary summing up the pivotal questions and the user-provided answers. This Sprint blueprint is readily available for users to save, share, or print, serving as a valuable reference guide for future endeavors and sustained progress (Figure 7).

***Gamified missions***

To enhance user engagement and facilitate the seamless completion of questions within each mission, we have introduced a scoring system tied to the quantity of questions. At the commencement of each mission, users receive information about the number of questions involved and the potential points they can earn. A dynamic point bar at the bottom of the screen continually updates, offering a visual representation of the user's score throughout the behavioral change journey. The cumulative score for the entire journey, upon completing all six missions, reaches a maximum of 100 points. This scoring mechanism adds a gamified element, motivating users to actively participate and progress through the missions.

### **Growth Section**

In the "Growth" section, users are afforded the opportunity to access a diverse range of sources, develop skills, and stay informed about events and bootcamps hosted by the Center for CHIIS.

### **Network Section**

Within the "Network" section, users are empowered to actively participate by joining groups, disseminating ideas, seeking support, and flourishing within our nurturing and collaborative learning community at CHIIS.
