# Supplementary material for: The Feasibility of AgileNudge+ Software to Facilitate Positive Behavioral Change: Mixed Methods Design
Source: JMIR Form Res. 2024 Nov 13;8:e57390. doi: 10.2196/57390 (PMC11602761; doi:10.2196/57390)
Supplement: Multimedia Appendix 4 [file formative_v8i1e57390_app4.doc]

## **Multimedia Appendix 4: Example of Nudge Design for a Recruitment Mindset Intervention**

### Participant 11 (P11), a research specialist for clinical trials, struggled with meeting recruitment targets on a monthly and weekly basis. Their goal was to improve recruitment outcomes while maintaining staff retention and engagement, as turnover and burnout rates were on the rise. P11 noted that the current recruitment process was yielding suboptimal results and required a refocus of the team's mindset. They aimed to shift recruitment behaviors to sustain better results over time, particularly since they lacked timely feedback in their current approach.

### **Step 1:** **Problem Identification**- P11 outlined their main challenges as follows:

### Current challenge: “Meeting recruitment targets on a monthly/weekly basis.”

### Desired outcome: “Successfully meeting target goals while maintaining staff engagement and avoiding turnover.”

### Behavioral changes required: P11 identified the need for a "reframing of mindset about the recruitment process" among the recruitment team.

### They noted that changing behavior was difficult due to a lack of timely feedback and consistent accountability and that any previous initiatives had been unsuccessful. To tackle these challenges, P11 set a project deadline of 2 weeks from initiation, with the team collectively dedicating 20 hours to finding a solution.

### **Step 2: Narrowing Target and Related Behaviors**- In the second phase, P11 refined the target behavior to focus on the mindset of the recruitment staff. The recruitment team frequently interacted with potential participants who were often “unhappy,” “skeptical,” “unmotivated,” or “overburdened.” P11 highlighted that improving the recruitment staff's mindset could enhance recruitment success. The only existing nudge that the team used was a "GPS tool", to track progress and interactions with participants. According to P11, it wasn’t achieving optimal results but did not seem to be detrimental to the process either.

### Target behavior: Recruitment staff needed to adjust their approach and recruitment mindset when interacting with potential participants.

### Related behavior(s): Recruitment staff struggled with timely, structured feedback and their recruitment mindsets, were not helping engage "skeptical" or "unmotivated" potential participants.

### **Step 3: Cognitive Bias Identification and Nudge Selection** - Using the Cognitive Bias Library within AgileNudge+, P11 identified "Ambiguity Bias" as a key factor in the recruitment process. Ambiguity bias occurs when individuals avoid options with uncertain outcomes. P11 believed that this bias explained the recruitment staff’s hesitation to follow through with participants who seemed disengaged, leading to missed recruitment opportunities. By restructuring the recruitment process to follow-up with potential participants regardless of initial assessments, the intention of the target nudge would be to limit the impact of the ambiguity bias on the recruitment staff.

### To change the behavior of the team when interacting with potential participants, P11 initially chose a nudge type related to "Priming and Salience" cognitive biases provided in Nudge Library to reframe recruitment calls. P11's goal was to ensure staff delivered simple and clear information to potential participants. By priming participants in the message delivery, the intention was to overcome initial negative judgments by participants. By increasing the salience of the message, participants would be more likely to attend to and remember study information.

### After scanning the Nudge Library within the software for related existing nudges, P11 selected an Information Provision nudge that provides a specific format to adjust how information is presented to potential participants. Specifically, the priming aspect incorporated was a preliminary screening question asked prior to asking for full participation (an example would be asking, “Do you experience lower back pain?” before recruiting for a study that intends to reduce lower back pain). Two key modifications were made to the script to increase salience: emphasizing the importance and impact of the study, and highlighting time and compensation early on. The nudge would be typified in an adjusted recruitment script that was given to all recruitment staff member, which would encourage staff to change their behavior. Additionally, rather than de-nudge the current GPS tool, P11 chose to add nudges into the GPS to help overcome the ambiguity bias. This included adding visual cues as simple follow-up triggers (i.e. a "Next Step" prompt for every potential participant, regardless of the initial assessment outcome) and a set of easy-to-follow, pre-define follow-up actions for participants who were rated as ‘uncertain’ or ‘neutral’ (i.e. buttons like "Send Check-In Email", "Schedule Phone Call", or "Request Missing Info").

### However, the initial EAST checklist score for the chosen nudge was 13/20, indicating that improvements were needed.

### AgileNudge+ provided EAST Checklist (Easy, Attractive, Social, Timely) with its explanation for P11 to use. As shown to P11, the EAST framework evaluates nudges based on four factors:

### Easy: How simple and straightforward the nudge is for staff to implement.

### Attractive: Whether the nudge is engaging and visually/mentally appealing.

### Social: If social factors, such as group norms, play a role in the nudge's design.

### Timely: Whether the nudge is delivered at the right time to maximize its impact.

### P11’s initial nudge design emotional appeal (Attractive) and got 2 out 5, while P11 believed that it is 3 out of 5 in terms of simplicity, Social and Timely factors got 4 out of 5. With the overall score of 13, AgileNudge+ guided P11 to revisit the nudge type as the score below 15 are less likely to succeed. To improve these areas, P11 made the following adjustments:

### Priming and Affect: The nudge type was redesigned to not only prime recruitment staff with simple and clear messages but also to emphasize emotional engagement (Affect). P11 was again prompted to review the existing literature for potential nudges and selecting further ways to modify the recruitment script and GPS dashboard. P11 elected to incorporate emotion-focused phrases into scripts that conveyed engagement (ie. “exciting opportunity,” “ground-breaking study,” or “revolutionary design”) and empathy for the participants’ burdens (i.e. "It’s perfectly normal to feel unsure, but we're here to provide all the information you need" or "I understand if you need time to think it over—this is a big decision, and we respect that"). Simple cues would also be added to the dashboards where progress was tracked (i.e. “Make it personal” or “Highlight the impact!”) to act as constant prompts for staff to include emotional elements in their conversations. By appealing to emotional drivers, such as the recruitment team’s motivation and empathy for the participants’ burdens, the nudge aimed to create a more compelling and engaging recruitment narrative. These changes raised the EAST score to 18/20, demonstrating a higher likelihood of effectively influencing recruitment staff behavior.

### MINDSPACE Checklist (Messenger, Incentives, Norms, Defaults, Salience, Priming, Affect, Commitments, Ego). AgileNudge+ takes a deeper dive into behavioral psychology and identifies nine key factors that drive decision-making and behavior change within MINDSPACE framework:

### Messenger: Who delivers the message and how their credibility influences acceptance.

### Incentives: The rewards or penalties that motivate behavior.

### Norms: The influence of social norms and peer behaviors.

### Defaults: The pre-set options that guide decisions.

### Salience: How attention-grabbing the nudge is.

### Priming: Subconscious cues that influence behavior.

### Affect: The emotional impact of the message.

### Commitments: The role of making commitments to driving behavior.

### Ego: How decisions affect self-image or pride.

### P11’s adjusted nudge type was also evaluated using this framework, focusing on several critical factors. After these adjustments, the nudge scored 37/50 on the MINDSPACE checklist, indicating an increased likelihood of success.

### **Step 4: Sprint Design and Testing**- P11 designed a sequential team sprint involving 4 recruitment staff members. The sprint’s objective was for staff to make a defined number of recruitments calls and for a specific success rate of calls in line with the study’s enrollment goals. Each staff member was responsible for making a set number of calls weekly, totaling 10 hours of work per week.

### P11 decided that the sprint should incorporate weekly data reviews and check-ins to maintain accountability, with performance being evaluated every week. The team would monitor their progress through REDCap, reviewing the number of recruitment calls made and their return on investment (ROI).

### **Step 5: Termination and Success Plan**- P11 defined clear success and termination metrics:

### Success: Continuity in recruitment calls over the next 90 days, with sustainable efforts from the recruitment staff. Enrollment rates to increase from 70% to 80% with no decrease in retention rates.

### Termination: If, after 5 weeks, the number of recruitment calls did not improve significantly, the sprint would be reevaluated, the nudge would be terminated, and a new strategy developed by P11.

### **Step 6: Creating a Standardized Minimally Viable Nudge for Diffusion**- If the nudge proved successful, P11 planned to create a Minimally Viable Nudge Procedure (MVP) for diffusion. This standardized approach would be integrated into the team’s workflow. Regular checks on adherence and buy-in from the recruitment team would ensure continual evaluation of long-term success and any adverse consequences from the nudge would be monitored, specifically any increase in staff burnout or turnover rates.

### If the nudge failed, P11 would evaluate probable causes for intervention failure, redefine the recruitment mindset, obtain more concrete buy-in from all recruiters, adjust recruitment hours, and dedicate more time to the nudge process. This reevaluation would help create a more focused and effective recruitment strategy.

### **Conclusion:** P11's structured approach to nudging recruitment behaviors highlights the importance of mindset shifts and timely feedback. By leveraging cognitive biases, adjusting nudge strategies, and creating a scalable framework, P11 implemented a data-driven, practical solution to meet recruitment targets, ultimately fostering a more engaged and productive recruitment team. The EAST and MINDSPACE checklists played a critical role in refining and optimizing the nudge, ensuring that the behavioral intervention aligned with psychological principles and practical application.
